# An ALKBH3 Inhibitor HUHS199 Suppresses Proliferation of Glioblastoma Cells by Targeting 1‐Methyladenosine on GADD45A mRNA

**DOI:** 10.1111/cbdd.70385

**Published:** 2026-09-01

**Authors:** Manami Yamada, Hiroaki Hase, Honoka Kitamura, Toshiya Morie, Shuntaro Aoi, Yuko Ueda, Kaori Kitae, Tatsuhiko Furukawa, Nayuta Higa, Ryosuke Hanaya, Kazutake Tsujikawa

**Affiliations:** ^1^ Laboratory of Stem Cell Regeneration and Adaptation, Graduate School of Pharmaceutical Sciences The University of Osaka Suita Osaka Japan; ^2^ Library Screening Center, Graduate School of Pharmaceutical Sciences The University of Osaka Suita Osaka Japan; ^3^ Drug Discovery Center, Graduate School of Pharmaceutical Sciences The University of Osaka Suita Osaka Japan; ^4^ Department of Pathology Kagoshima University Graduate School of Medical and Dental Sciences Kagoshima Japan; ^5^ Department of Neurosurgery Kagoshima University Graduate School of Medical and Dental Sciences Kagoshima Japan

**Keywords:** 1‐methyladenosine, ALKBH3, GADD45A, glioblastoma, RNA demethylation, small compound inhibitor

## Abstract

Glioblastoma (GBM) is a highly aggressive, poor‐prognosis brain tumor classified as WHO grade IV, for which effective treatments remain limited. Although AlkB homolog 3 (ALKBH3), a demethylase for 1‐methyladenosine (m1A) and 3‐methylcytidine (m3C) in DNA and RNA, has been implicated in cancer cell proliferation, its functional role in GBM remains unclear. We aimed to characterize the role of ALKBH3 in GBM and develop and evaluate a patented ALKBH3 inhibitor, HUHS199, to identify its molecular targets and therapeutic potential. We found that ALKBH3 was highly expressed in clinical GBM specimens; its knockdown inhibited GBM cell proliferation. HUHS199 inhibited GBM cell proliferation in a dose‐dependent manner, induced G1 phase cell cycle arrest, and increased m1A levels in RNA. Furthermore, RNA immunoprecipitation–microarray analysis using an anti‐m1A antibody identified growth arrest and DNA‐damage‐inducible protein GADD45 alpha (GADD45A) mRNA as a target of ALKBH3‐mediated demethylation. These findings suggest that ALKBH3 promotes GBM cell proliferation via m1A demethylation of *GADD45A* mRNA. Collectively, this study highlights the therapeutic potential of ALKBH3 inhibition and presents HUHS199 as a first‐in‐class candidate for GBM treatment.

## Graphical abstract

HUHS199, a novel inhibitor of ALKBH3, suppresses glioblastoma cell proliferation, induces G1 cell cycle arrest, and increases RNA m1A levels, with concomitant changes in GADD45A mRNA methylation status, highlighting the pathological relevance of ALKBH3‐mediated RNA demethylation in glioblastoma.

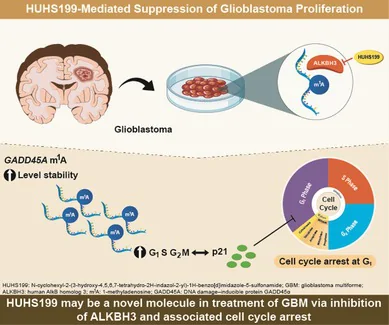

**Abbreviations**
ALKBH: human AlkB homolog
GADD45A: DNA‐damage‐inducible protein GADD45 alpha
GBM: glioblastoma multiforme
HUHS015: 1‐(5‐methyl‐1H‐benzimidazol‐2‐yl)‐4‐benzyl‐3‐methyl‐1H‐pyrazol‐5‐ol
HUHS199: N‐cyclohexyl‐2‐(3‐hydroxy‐4,5,6,7‐tetrahydro‐2H‐indazol‐2‐yl)‐1H‐benzo[d]imidazole‐5‐sulfonamide
m1A: 1‐methyladenosine
m3C: 3‐methylcytidine
m6A: N^6^‐methyladenosine
ssDNA: single‐stranded DNA

## 1 Introduction

Glioblastoma (GBM) is the most common malignant primary tumor of the central nervous system, accounting for approximately 14.5% of all primary brain tumors and 48% of malignant primary brain tumors (Ostrom et al. 2018). Classified as Grade IV, the highest grade of malignancy according to the WHO classification system, it is an extremely poor‐prognosis brain tumor characterized by high invasiveness and recurrence rates (Wen and Reardon 2016; Tan et al. 2020). The current standard treatment for GBM involves multidisciplinary therapy, which combines surgical resection, radiation therapy, and chemotherapy; however, even with optimal treatment, the median survival remains at approximately 15 months for patients with GBM (Omuro and DeAngelis 2013; Ohgaki and Kleihues 2005; Stupp et al. 2009). Despite recent advances in identifying various signaling pathways, transcription factors, and microRNAs involved in GBM initiation and progression, therapeutic strategies targeting these elements have not significantly improved patient outcomes (Stringer et al. 2016; Liu et al. 2020; Lee et al. 2014; Banelli et al. 2017; Wang and Ma 2015). Therefore, elucidating novel molecular mechanisms underlying the malignant progression of GBM and identifying novel therapeutic targets are urgently necessary.

The *E. coli* protein AlkB, a 2‐oxoglutarate‐ and Fe(II)‐dependent oxygenase, repairs DNA alkylation damage by the oxidative demethylation of 1‐methyladenosine (m1A) and 3‐methylcytidine (m3C) in DNA (Aas et al. 2003; Falnes et al. 2002; Trewick et al. 2002). In humans, nine AlkB homolog (ALKBH) family members (ALKBH1–ALKBH8 and FTO) have been identified, each with different substrate specificities, catalyzing methylation modifications in DNA, RNA, and proteins (Kurowski et al. 2003; Tsujikawa et al. 2007; Jia et al. 2011; Yang et al. 2025). For example, ALKBH2 repairs DNA alkylation damage by demethylating m1A and m3C in double‐stranded DNA (Aas et al. 2003). ALKBH4 catalyzes the demethylation of actin, thereby regulating actin–myosin II interactions (Li et al. 2013). ALKBH5 and FTO are primarily recognized as “erasers” of N^6^‐methyladenosine (m6A) on mRNA; ALKBH5 functions as an RNA modification regulator to demethylate m6A in mRNA nuclei (Zheng et al. 2013), whereas FTO demethylates m6A and N^6^,2′‐O‐dimethyladenosine (m6Am) (Mauer et al. 2017). ALKBH family members have attracted considerable attention in cancer. In GBM, ALKBH2 is highly expressed; its overexpression confers resistance to the DNA‐alkylating agent temozolomide (Johannessen et al. 2013). ALKBH5 enhances the expression of FOXM1 by demethylating its nascent transcripts (Zhang et al. 2017).

ALKBH3 was initially identified as a gene highly expressed in prostate cancer (Konishi et al. 2005). Subsequent studies have reported elevated ALKBH3 expression in various cancers, including pancreatic cancer, lung cancer, and urothelial carcinoma, compared with that in adjacent normal tissues; its overexpression has been associated with poor prognosis. (Yamato et al. 2012; Hotta et al. 2015; Wang et al. 2018; Gu et al. 2024; Konishi et al. 2005; Tasaki et al. 2011; Shimada et al. 2012). Functional analyses have demonstrated that ALKBH3 is an enzyme that oxidatively demethylates m1A and m3C in alkylation‐damaged single‐stranded DNA (ssDNA) and RNA (Trewick et al. 2002; Aas et al. 2003; Ougland et al. 2004; Dango et al. 2011). Alkylated ssDNA is first unwound by ASCC3, a DNA helicase that associates with ALKBH3, after which ALKBH3 demethylates its m1A sites. ALKBH3 also demethylates m1A in tRNA, thereby enhancing protein translation efficiency (Ueda et al. 2017). Furthermore, ALKBH3 plays a crucial role in maintaining cellular survival, proliferation, and protein synthesis in colorectal and lung cancer cells (Luo et al. 2009; Tasaki et al. 2011). In contrast, no significant phenotypes have been observed in ALKBH3 knockout mice (Ringvoll et al. 2006). These findings suggest that ALKBH3 is a promising therapeutic target for cancer; selective ALKBH3 inhibitors may serve as novel, clinically effective anticancer agents with reduced toxicity and side effects.

Currently, 1‐(5‐methyl‐1H‐benzimidazol‐2‐yl)‐4‐benzyl‐3‐methyl‐1H‐pyrazol‐5‐ol (HUHS015; Figure S1A) is known as a low‐molecular‐weight inhibitor of ALKBH3 demethylase activity (Nakao et al. 2014; Mabuchi et al. 2015; Ueda et al. 2018). HUHS015 potently inhibited ALKBH3 demethylase activity and significantly suppressed the proliferation of prostate cancer cells (DU145) in vitro (IC_50_ = 2.2 μM) and in vivo (32 mg/kg, subcutaneous administration) in a mouse xenograft model. In this study, we investigated the expression and function of ALKBH3 in GBM. Furthermore, through the derivative synthesis of HUHS015, we developed a newly designed compound, HUHS199, that exhibited a relatively potent inhibition of ALKBH3 enzyme activity, evaluated its effects in human GBM cells, and identified ALKBH3 substrates.

## 2 Materials and Methods

### 2.1 Recombinant Human ALKBH3

Recombinant FLAG‐His‐tagged human ALKBH3 (hALKBH3) was produced by Sysmex (Osaka, Japan), as described previously (Ueda et al. 2016). Briefly, 20 silkworm pupae were infected with the hALKBH3 recombinant baculovirus. Six days post‐infection, the pupae were homogenized and lysed. The ultracentrifugation supernatant was subjected to affinity and ion exchange purifications and then dialyzed into 20 mM Tris–HCl (pH 8.0), 150 mM NaCl, 50% (w/v) glycerol, and 1 mM dithiothreitol.

### 2.2 Human Specimens

Twenty‐seven samples of cancerous tissues and adjacent non‐cancerous tissues were collected from patients with *IDH*‐wildtype GBM (age 44–82 years, mean age: 67 years; 16 men (64%) and 9 women (36%)) who underwent resection surgery at Kagoshima University Hospital (Japan). Written consent was obtained from each patient for the use of postoperative specimens for the purposes of this study. This study was conducted in accordance with the principles of the Declaration of Helsinki and was approved by the Ethics Committee on Epidemiological Studies of the Graduate School of Medical and Dental Sciences, Kagoshima University (approval number: 180035 Eki‐Kai 6) and the Research Ethics Review Committee of the Graduate School of Pharmaceutical Sciences, The University of Osaka (approval number: yakuhito30‐4‐4).

### 2.3 Cell Culture

Human GBM cell lines, U251MG and LN229, were obtained from the American Type Culture Collection (ATCC). U251MG was cultured in EMEM medium containing 10% heat‐inactivated fetal bovine serum (FBS; Thermo Fisher Scientific, Waltham, Massachusetts, USA) and 100 μg/mL kanamycin (Wako Pure Chemical Industries, Tokyo, Japan). LN229 cells were cultured in high‐glucose DMEM medium (Wako Pure Chemical Industries) supplemented with 5% FBS (Thermo Fisher Scientific) and 100 μg/mL kanamycin. Cells were cultured at 37°C in a 5% CO_2_ atmosphere.

### 2.4 RNA Isolation and m1A RNA Immunoprecipitation

Total RNA was purified using the TRIzol reagent (Invitrogen, Waltham, MA, USA) according to the manufacturer's protocol. RNA immunoprecipitation was performed using a microRNA RIP assay kit (Medical & Biological Laboratories Co. Ltd., Tokyo, Japan) according to the manufacturer's instructions. The obtained RNA was incubated with an anti‐m1A monoclonal antibody (D345‐3; MBL, Japan) conjugated to Dynabeads Protein G (Invitrogen). The bound RNA‐anti‐m1A monoclonal antibody complex was washed with kit buffer and ice‐cold ethanol. Subsequently, RNA bound to the beads was eluted from the Dynabeads using nuclease‐free water.

### 2.5 Quantitative Analysis of RNA Modification Levels

Quantitative analysis of the RNA modification levels was performed using the method described in a previous study (Kogaki et al. 2021). Briefly, 200 ng of RNA samples from 27 matched pairs of cancerous and adjacent non‐cancerous tissue from postoperative GBM tissues was mixed with 2 ng of 2′‐deoxyguanosine‐N^15^5 (dG15N5) as an internal standard and treated with nuclease P1 (Wako Pure Chemical Industries; 145‐08221) at 45°C for 2 h. Ribonucleotides were then treated with bacterial alkaline phosphatase (Takara Bio Inc., Shiga, Japan; 2120A) at 37°C for 2 h. Nucleoside content was measured with a Xevo TQ‐XS UHPLC‐UniSpray‐MS/MS (Waters Corporation, Milford, MA, USA). Each nucleoside was quantified from the calibration curve using the ratio of the peak area to the internal standard. The ratio of modified nucleosides to unmodified nucleosides was calculated based on the calibration concentration. All peaks were automatically integrated using the MASSLYNX XS software (Waters Corporation, Massachusetts, USA). Nucleosides were quantified from the calibration curve using the ratio of each peak area to the peak area of dG15N5 (the internal standard).

### 2.6 ALKBH3 Knockdown by siRNA Transfection

Cells (1.5 × 10^5^) were seeded in a six‐well plate and transfected with either 10 nM control siRNA (Silencer Select Negative Control No. 1, #4390844; Invitrogen) or ALKBH3 siRNAs (s47969 and s225888; Ambion, Austin, Texas, USA) using Lipofectamine RNAiMAX Transfection Reagent (Invitrogen), following the manufacturer's protocol. The sequences of siRNAs targeting ALKBH3 were as follows: s47969; sense, CCUCAGCAGGUAGUACGUAtt; antisense, UACGUACUACCUGCUGAGGtt; s225888; sense, CAGAGAGGAUAUAACUUAUtt; antisense, AUAAGUUAUAUCCUCUCUGat.

### 2.7 ELISA Evaluation of ALKBH3 Demethylase Activity

Approximately 150 fmol/well of m1A‐containing biotinylated oligonucleotide RNA (biotin‐UUUGGACUUGGUUGG_m1A_CUUU; Genedesign, Osaka, Japan) or 100 fmol/well of m1A‐containing biotinylated oligonucleotide DNA (biotin‐TTTGGACTTGGTTGG_m1A_CTTT) was incubated in a streptavidin‐coated plate (NUNC, Waltham, MA, USA; 436014) at room temperature for 2 h. Meanwhile, in the demethylation enzyme reaction buffer (50 mM HEPES [pH 7.3 ± 0.1], 1 mM ascorbic acid, 100 μM 2‐oxoglutaric acid, 40 μM FeSO_4_·7H_2_O), 25 ng recombinant hALKBH3 and HUHS199 were pre‐incubated at room temperature for 30 min. After washing the plate with 2 × SSCT buffer (Wako), the pre‐incubated reaction solution was added; the demethylation enzyme reaction was performed at 26°C for 1 h. After washing the plate with phosphate‐buffered saline (PBS)‐Tween 20, 0.1 μg/mL anti‐m1A monoclonal antibody (MBL, D345‐3) was added, followed by horseradish peroxidase‐linked anti‐mouse IgG (1:3000, Cell Signaling Technology, Danvers, MA, USA, 7076S). The detection reagent used was The ABTS microwell peroxidase substrate system (KPL, Milford, MA, USA, 5120‐0032). An enzyme reaction calibration curve was constructed using recombinant hALKBH3 at final concentrations of 40, 35, 30, 25, 20, 15, 10, and 0 ng.

### 2.8 Cell Proliferation Assay

Cells (2 × 10^3^/well) were seeded in 96‐well plates before control DMSO or HUHS199 addition and cultured in a humidified atmosphere of 5% CO_2_ at 37°C. Subsequently, the cells were incubated with 100 μL medium containing 10 μL CCK‐8 cell counting kit (Dojindo, Kumamoto, Japan) at 37°C for 2 h. Cell viability was quantified by measuring absorbance at 450 nm using a Multiskan FC microplate reader (Thermo Fisher Scientific).

### 2.9 Real‐Time Cell Proliferation Analysis

The cells were detached 24 h post‐transfection and reseeded at 500 cells/well in an E‐Plate PET (ACEA Biosciences, San Diego, CA, USA). The proliferation capacity was assessed using xCELLigence DP (ACEA Biosciences) by measuring the electrical resistance at the bottom of the plate.

### 2.10 Cell Cycle Analysis

Cells (1 × 10^5^ cells/well) were seeded in six‐well plates and treated with different concentrations of HUHS199 for 24 h. The cells were collected, washed three times with cold PBS, and fixed in 70% ice‐cold ethanol. The cells were then washed twice with cold PBS and treated with 1.5 mg/mL RNase (NIPPON GENE, Tokyo, Japan) solution and 50 μg/mL propidium iodide (PI; Sigma‐Aldrich, St. Louis, MO, USA) for 30 min each. Cell suspensions were passed through a nylon mesh filter, measured using a ZE5 Cell Analyzer (Bio‐Rad, Hercules, CA, USA), and analyzed using FlowJo v10 (Becton Dickinson, Franklin Lakes, NJ, USA).

### 2.11 Microarray Analysis

U251MG cells were seeded in a 10‐cm dish and treated with 10 μM HUHS199 or ALKBH3 siRNA #2 for 24 h. Total RNA was extracted from the harvested cells using the TRIzol reagent. The RNA concentration was measured with a NanoDrop microvolume spectrophotometer (Thermo Fisher Scientific). RNA quality was assessed using an Experion RNA StdSens Analysis Kit and Experion Automated Electrophoresis System (Bio‐Rad). cDNA was synthesized from 100 ng total RNA. Following fragmentation, cDNA was biotinylated using a GeneChip WT PLUS Reagent Kit (Thermo Fisher Scientific) and hybridized to the human Clariom S array chip (Thermo Fisher Scientific) at 45°C for 16 h using a GeneChip Hybridization, Wash, and Stain Kit (Thermo Fisher Scientific). The array chip was washed and stained with a streptavidin‐phycoerythrin conjugate on a GeneChip Fluidics Station (Thermo Fisher Scientific). Fluorescence signals from the array were measured using a GeneChip Scanner 3000 (Thermo Fisher Scientific). Raw array data were normalized and summarized using the Signal Space Transformation‐Robust Multi‐array Analysis (SST‐RMA) algorithm; quality assessment was performed using the Transcriptome Analysis Console (TAC) software version 4.0.3.14 (Thermo Fisher Scientific). Probe annotation was performed using the human reference genome (GRCh38). Gene expression levels and false discovery rate–corrected *p*‐values (FDR *p*‐values) were calculated using TAC software.

### 2.12 mRNA Expression Analysis

cDNA was synthesized from the RNA samples using a PrimeScript RT Reagent Kit (Takara Bio Inc.). Quantitative PCR (qPCR) was performed with THUNDERBIRD SYBR qPCR Mix (Toyobo Co. Ltd., Osaka, Japan) on a CFX96 Connect Real‐Time System (Bio‐Rad). The primers for qRT‐PCR of *ALKBH3*, *p21*, *GADD45A*, and *EEF1A1* are listed: *ALKBH3*, 5′‐TGCCACACGCACATTTGAGA‐3′ and 5′‐ATGATTAACAAGGTCCCATGATCCA‐3′; *p21*, 5′‐ACCTCTCAGGGCCGAAAAC‐3′ and 5′‐TAGGGCTTCCTCTTGGAGAA‐3′; *GADD45A*, 5′‐CGAGAACGACATCAACATCC‐3′ and 5′‐GATCCATGTAGCGACTTTCC‐3′; *EEF1A1*, 5′‐GTATTGGATTGCCACACGGC‐3′ and 5′‐CAAAGCGACCCAAAGGTGGA‐3′. The mRNA expression levels assessed by qPCR were normalized using *eukaryotic translation elongation factor 1 alpha 1* (*EEF1A1*) as an internal standard.

### 2.13 Immunoblotting Analysis

Immunoblotting was performed using the JESS capillary electrophoresis system (ProteinSimple, San Jose, CA, USA). Cells treated with HUHS199 for 24 h were lysed in RIPA buffer (Thermo Fisher Scientific) containing 1% protease inhibitor to obtain lysates. The lysates were loaded onto a Jess separation module (12–230 kDa). The primary antibodies used were anti‐p21 Waf1/Cip1 antibody (1:200, Cell Signaling Technology, 2946S) and anti‐EEF1A1 (1:20, Proteintech, Rosemont, IL, USA; 67495‐1‐Ig). Proteins were detected and analyzed using the Compass for Simple Western software (ProteinSimple).

### 2.14 Flow Cytometric Analysis of Intracellular m1A Levels

Cells were fixed for 20 min with BD Cytofix/Cytoperm (Becton Dickinson) 24 h after HUHS199 treatment and washed with BD 1 × Perm/Wash Buffer (Becton Dickinson). Samples were incubated at 4°C for 1 h with anti‐m1A antibody (1:700, MBL, D345‐3), followed by incubation at 4°C for 30 min with Alexa Fluor 488 goat anti‐mouse IgG antibody (1:1000, Invitrogen, A11029). After staining, the fluorescence intensity of FITC (detection wavelength: 525 nm) was analyzed using a MACSQuant (Miltenyi Biotec, Bergisch Gladbach, Germany).

### 2.15 m1A Immunofluorescence and Confocal Microscopy

Cells were treated with HUHS199 for 24 h, fixed with 4% formaldehyde for 1 h, and permeabilized with 0.2% Triton X‐100 for 5 min. Cells for RNase treatment were treated with 5 mg/mL RNase solution for 1 h, followed by blocking with 5% skim milk for 1 h. Samples were incubated overnight at 4°C with anti‐m1A antibody, followed by incubation with Alexa Fluor 568 goat anti‐mouse IgG antibody (Invitrogen, A11031). DAPI Fluoromount‐G (Southern Biotech, Birmingham, AL, USA), which allows the visualization of DNA content, was used as the mounting medium. Imaging was performed using a Nikon Ti2‐E based confocal laser scanning microscope (Nikon, Tokyo, Japan) with a 60× objective lens. Representative regions of interest (ROIs) were visualized at relatively high digital magnification in the NIS‐Elements software (Nikon) and exported for figure preparation. Images were acquired for all the samples using identical laser output and detector gain settings. Fluorescence intensity profiles were created with the NIS‐Elements software (Nikon) by manually setting ROIs on the cells and measuring the fluorescence intensity.

### 2.16 mRNA Stability Assay

To analyze the degradation rate of DNA‐damage‐inducible protein GADD45 alpha (*GADD45A*) mRNA in LN229 cells, 2 × 10^5^ cells were incubated with 10 μM HUHS199 and 1 mg/mL actinomycin D (Wako). Cells were harvested at 2, 4, 6, 8, 10, 12, and 24 h after treatment; RNA extraction and cDNA synthesis were performed. Untreated cells served as the time 0 control.

### 2.17 Analysis of Publicly Available GBM Dataset

The Cancer Genome Atlas (TCGA) microarray data (Agilent‐4502A) from adult patients with GBM were analyzed using the GlioVis portal (http://gliovis.bioinfo.cnio.es) (accessed on March 3, 2026). For hazard ratios, we downloaded the same TCGA dataset used in GlioVis and calculated them with the Cox proportional hazards regression model implemented in the R software (version 4.4.1), with the low‐expression group serving as the reference group.

### 2.18 Statistical Analysis

Statistical analyses and visualization of data were performed using GraphPad Prism (GraphPad Prism 10, GraphPad Software). Student's *t*‐test or one‐way ANOVA with post hoc Tukey's test was used to assess the quantitative data. For between‐group statistical comparisons, the Wilcoxon signed‐rank test was used for the paired samples. Statistical significance was set at *p* < 0.05.

## 3 Results

### 3.1 High *ALKBH3* Expression in GBM Correlates With Poor Prognosis

**FIGURE 1:**
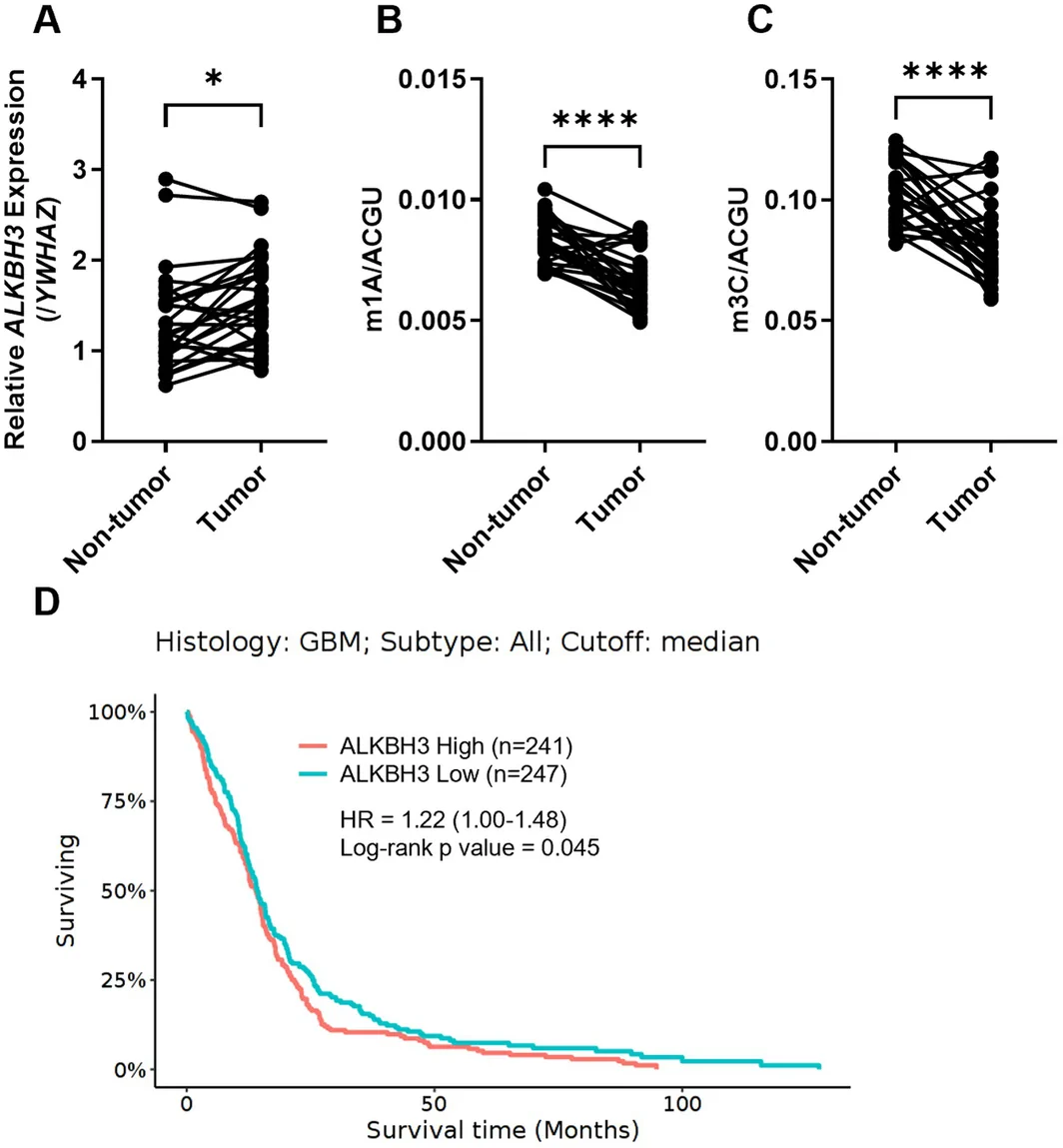
High ALKBH3 expression in patients with GBM is associated with decreased levels of m1A and m3C, as well as poor prognosis. (A) ALKBH3 expression levels and (B) m1A and (C) m3C levels of RNA in the tumor and non‐tumor regions of matched‐pair postoperative GBM specimens (*n* = 27); paired *t*‐test: *p < 0.05, ****p < 0.0001. (D) Overall survival time for patients with GBM (ALKBH3 high, *n* = 241; ALKBH3 low, *n* = 247) was analyzed by Kaplan–Meier curve comparison using the GlioVis portal.

*ALKBH3* expression in matched‐pair postoperative GBM specimens was quantified using qPCR. *ALKBH3* expression was significantly higher in GBM tumor tissues than in adjacent non‐tumor tissues (Figure 1A). Analysis of the TCGA database indicated that patients with GBM with high *ALKBH3* expression had poor prognosis (Figure 1B). Furthermore, when GBM was classified into three subtypes (classical, mesenchymal, and proneural), no correlation between *ALKBH3* overexpression and prognosis was observed in the classical subtype. However, a significant correlation between *ALKBH3* expression and poor prognosis was observed in the mesenchymal and proneural subtypes (Figure S1A). ALKBH3 is an enzyme that oxidatively demethylates m1A and m3C in RNA. Therefore, total RNA was extracted from GBM specimens, enzymatically digested into nucleosides; m1A and m3C levels were measured using mass spectrometry. The results confirmed that the levels of m1A (Figure 1C) and m3C (Figure 1D) were significantly reduced in tumor tissues compared with those in adjacent non‐tumor tissues.

### 3.2 Knockdown of *ALKBH3* Suppresses Proliferation of GBM Cells

**FIGURE 2:**
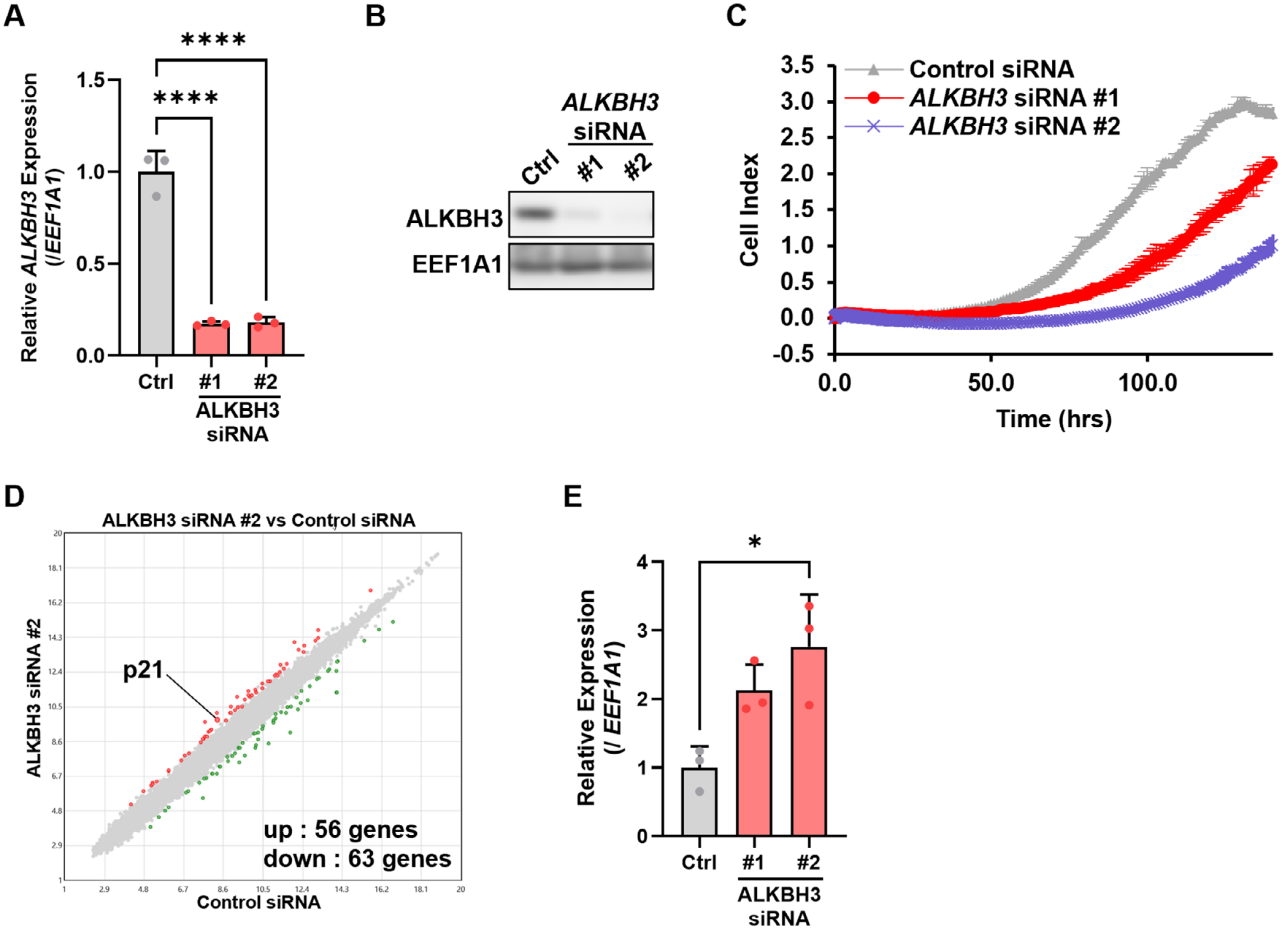
*ALKBH3* knockdown suppressed GBM cell proliferation. (A) *ALKBH3* mRNA levels were analyzed by qPCR in control U251MG cells (siControl) or *ALKBH3*‐knockdown U251MG cells (siALKBH3). Values are shown as mean ± S.D. (*n* = 3); one‐way ANOVA post hoc Bonferroni's test: ****p < 0.0001. (B) Western blot analysis of whole cell lysates prepared from control U251MG cells (siControl) or ALKBH3‐knockdown U251MG cells (siALKBH3) using anti‐ALKBH3 and anti‐EEF1A1 antibodies. Representative images from three independent experiments are shown. (C) Evaluation of the proliferative potential of *ALKBH3*‐knockdown U251MG cells using a real‐time cell analysis system. The data shows representative cell proliferation curves from duplicate experiments. (D) Microarray analysis identified genes whose expression increased (red) and decreased (green) in ALKBH3‐knockdown U251MG cells. (E) qRT‐PCR analysis of *p21* mRNA levels in ALKBH3‐knockdown U251MG cells. Values are shown as mean ± S.D. (*n* = 3); one‐way ANOVA post hoc Bonferroni's test: *p < 0.05.

To investigate the role of ALKBH3 in GBM cell proliferation, we performed knockdown experiments using *ALKBH3*‐specific siRNA. The human GBM cell line U251MG was transfected with two types of siRNAs targeting different sequences of the *ALKBH3* gene, resulting in a significant reduction in ALKBH3 expression at mRNA and protein levels (Figure 2A,B). Proliferation of ALKBH3‐knockdown U251MG cells was assessed in real time using the xCELLigence system based on electrical resistance. Both *ALKBH3*‐targeting siRNAs markedly suppressed the proliferation of U251MG cells (Figure 2C). Similar suppressive effects of *ALKBH3* siRNAs were also observed in another GBM cell line, LN229 (Figure S2A–C). To further elucidate the mechanism underlying proliferation inhibition by *ALKBH3* knockdown, we performed microarray analysis. The results identified p21 as one of the genes whose expression was upregulated upon transfection with *ALKBH3* siRNA #2 (Figure 2D). We then verified *p21* mRNA expression by qRT‐PCR and found that *p21* expression was significantly increased in cells transfected with ALKBH3 siRNA #2 (Figure 2E). An increase in *p21* expression was also observed in LN229 cells, with a significant increase detected for *ALKBH3* siRNA #2 (Figure S2D).

### 3.3 ALKBH3 Demethylase Inhibitor HUHS199 Suppresses GBM Cell Proliferation by Inducing G1 Cell Cycle Arrest

**FIGURE 3:**
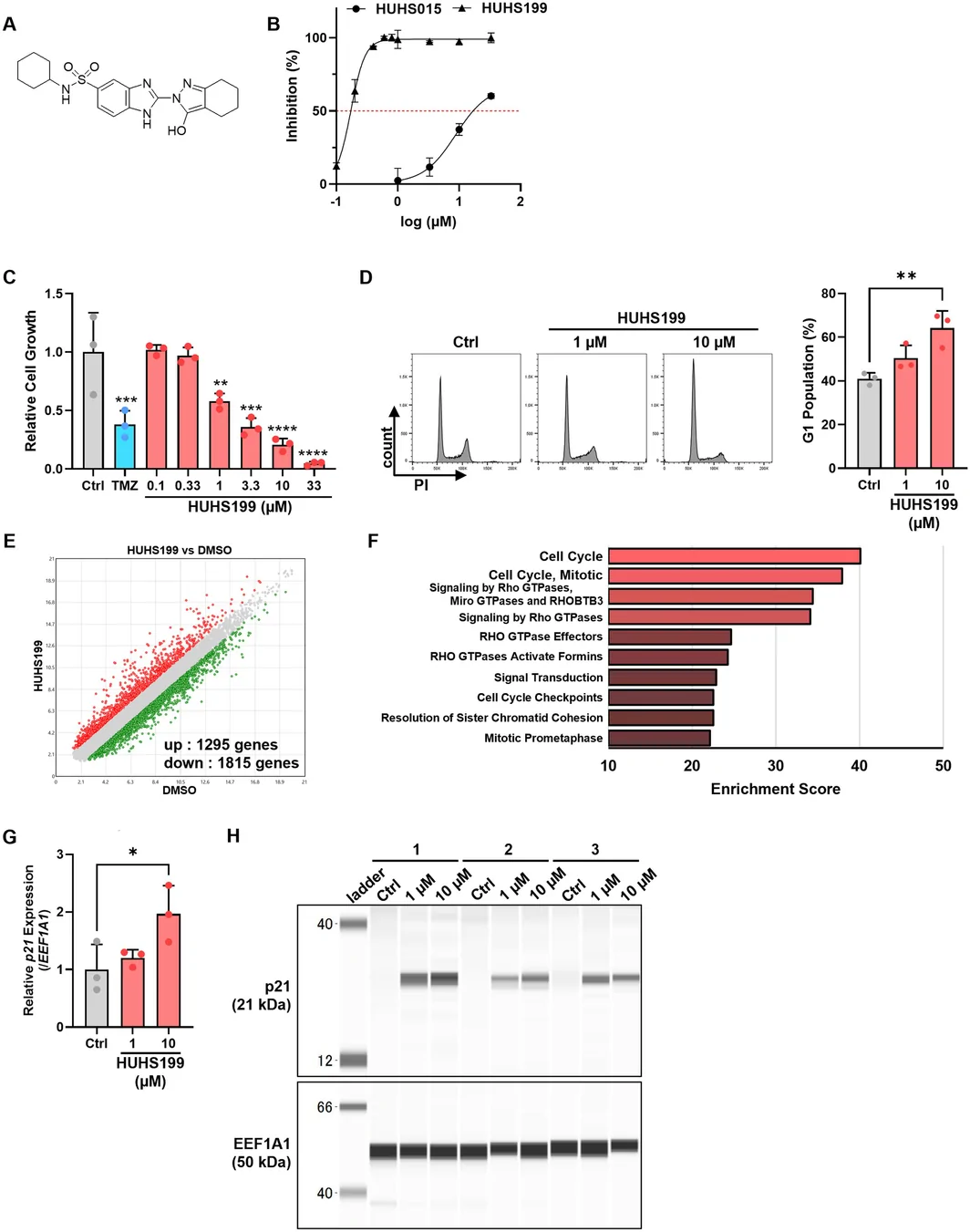
HUHS199 inhibits the proliferation of U251MG cells via G1 arrest of cell cycle. (A) Chemical structure of HUHS199. (B) Comparison of the inhibitory effects of HUHS199 and HUHS015 on ALKBH3 demethylase activity using a proprietary ELISA method employing the m1A‐containing RNA oligonucleotide as a substrate. (C) Inhibitory effect of HUHS199 on the proliferation of U251MG cells. HUHS199 and temozolomide (TMZ; used as a positive control) were added to U251MG cells and cultured for 2 days. Values are shown as mean ± S.D. (*n* = 3); one‐way ANOVA post hoc Bonferroni's test: **p < 0.01, ***p < 0.001, ****p < 0.0001. (D) Cell cycle analysis of U251MG cells treated with HUHS199 by flow cytometry. Values are shown as mean ± S.D. (*n* = 3); one‐way ANOVA post hoc Bonferroni's test: **p < 0.01. (E) Microarray analysis identified genes whose expression increased (red) and decreased (green) in U251MG cells upon HUHS199 addition. (F) Enrichment analysis of genes whose expression decreased upon HUHS199 addition. (G) qPCR analysis of *p21* mRNA levels in U251MG cells upon HUHS199 addition. Values are shown as mean ± S.D. (*n* = 3); one‐way ANOVA post hoc Bonferroni's test: *p < 0.05. (H) Whole cell lysates prepared from U251MG cells treated with HUHS199 were analyzed by JESS capillary western blotting using anti‐p21 and anti‐EEF1A1 antibodies.

Given that ALKBH3 knockdown inhibited GBM cell proliferation, we next evaluated HUHS199, an inhibitor of ALKBH3 enzymatic activity (Figure 3A). HUHS199 is a newly designed compound derived from the parent structure of HUHS015 (Nakao et al. 2014) (Figure S3A) through derivative synthesis (Figure S3B). The inhibitory effect of HUHS199 on ALKBH3 demethylase activity was first examined using an ELISA‐based assay with the silkworm‐derived recombinant ALKBH3. In the ELISA system, RNA oligonucleotides containing m1A were used as substrates; the residual m1A content after demethylation was determined using an anti‐m1A antibody. In this ELISA system, HUHS199 (IC_50_ = 0.17 μM) more potently inhibited the RNA demethylase activity of ALKBH3 than HUHS015 (IC_50_ = 8.79 μM) (Figure 3B). Moreover, HUHS199 exhibited significant inhibitory activity against ALKBH3 demethylase in an ELISA using m1A‐containing DNA oligonucleotides as substrates (Figure S3C). The antiproliferative effect of HUHS199 on U251MG GBM cells was evaluated using the CCK‐8 assay. HUHS199 significantly inhibited cell proliferation in a concentration‐dependent manner (Figure 3C). Similar proliferation‐inhibitory effects of HUHS199 were observed in LN229 and U87MG cells (Figure S4A). Conversely, the effect of HUHS199 on the human normal keratinocyte cell line PSVK1 was tenfold weaker than that on GBM cells (Figure S4B). The effect of HUHS199 on the cell cycle was examined using flow cytometry. HUHS199‐treated U251MG cells showed an increased proportion of cells in the G1 phase, indicating induction of G1 arrest (Figure 3D). G1 phase cell cycle arrest was similarly observed in LN229 and U87MG cells after the addition of HUHS199 (Figure S4C). To investigate the mechanism underlying the HUHS199‐induced inhibition of proliferation in U251MG cells, microarray analysis was performed. We identified 1295 genes whose expression increased and 1815 genes whose expression decreased upon HUHS199 addition (Figure 3E). Gene set enrichment analysis revealed that the cell cycle–related pathways were suppressed in HUHS199‐treated U251MG cells (Figure 3F). G1 cell cycle arrest is induced by p21 expression. *p21* gene expression was determined by qPCR; a significant increase in *p21* expression was observed in HUHS199‐treated U251MG cells compared with that in control cells (Figure 3G). Increased p21 protein expression was confirmed by JESS capillary western blotting (Figure 3H). Similarly, in HUHS199‐treated LN229 cells, p21 expression increased in a dose‐dependent manner (Figure S4D–E).

### 3.4 HUHS199 Increases m1A Levels of Cytoplasmic RNA in GBM Cells

**FIGURE 4:**
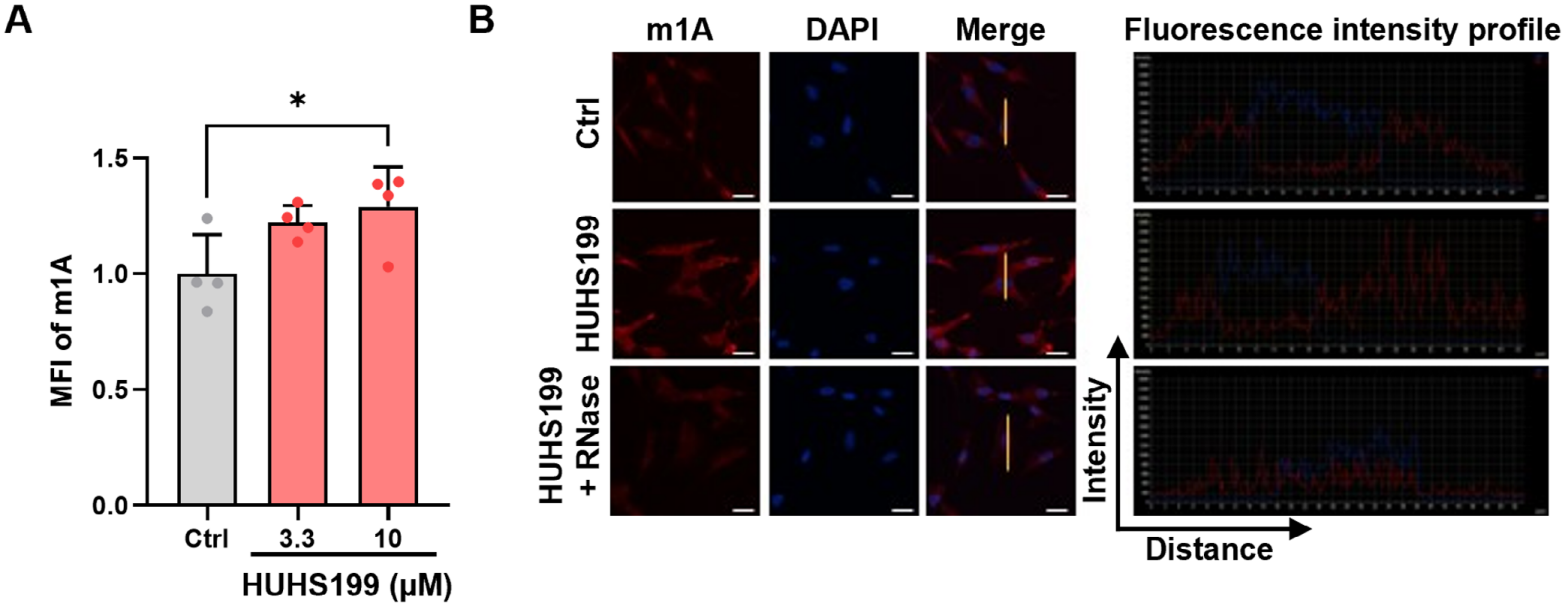
HUHS199 increases m1A levels of RNA in U251MG cells. (A) Evaluation of m1A levels in U251MG cells upon HUHS199 addition by flow cytometry. U251MG cells treated with control or HUHS199 were fixed and stained with anti‐m1A antibody. The average fluorescence intensity measured within the cells was expressed as mean fluorescence intensity (MFI). Values are shown as mean ± S.D. (*n* = 3); one‐way ANOVA post hoc Bonferroni's test: *p < 0.05. (B) Immunocytochemical evaluation of intracellular m1A levels in U251MG cells upon HUHS199 (10 μM) addition. Each profile (red: M1A, blue: DAPI) shows the fluorescence intensity distribution along the yellow line indicated in the image. Images were acquired with a 60× objective lens. Scale bars (white): 20 μm. Representative images from three independent experiments are shown.

To determine whether the growth‐inhibitory effect of HUHS199 on GBM cells was mediated through the inhibition of ALKBH3 RNA demethylase activity, m1A levels were quantified in HUHS199‐treated U251MG cells by flow cytometry using an anti‐m1A antibody. HUHS199 treatment significantly increased m1A levels in U251MG cells compared with control cells (Figure 4A). Similar experiments using LN229 cells revealed an increasing trend in m1A expression (Figure S5A). Furthermore, fluorescence immunostaining of U251MG cells with an anti‐m1A antibody confirmed increased fluorescence intensity following HUHS199 addition. The increase in m1A levels was predominantly localized in the cytoplasm; RNase treatment prior to cellular staining with the anti‐m1A antibody significantly attenuated the m1A signal. The characteristics and changes in intracellular signal intensity were also confirmed by fluorescence intensity profiling (Figure 4B). Similar results were observed in LN229 cells (Figure S5B). These findings suggest that the increase in m1A levels induced by HUHS199 treatment originates primarily from cytoplasmic RNA.

### 3.5 ALKBH3 Regulates the m1A Level of *GADD45A* mRNA

**FIGURE 5:**
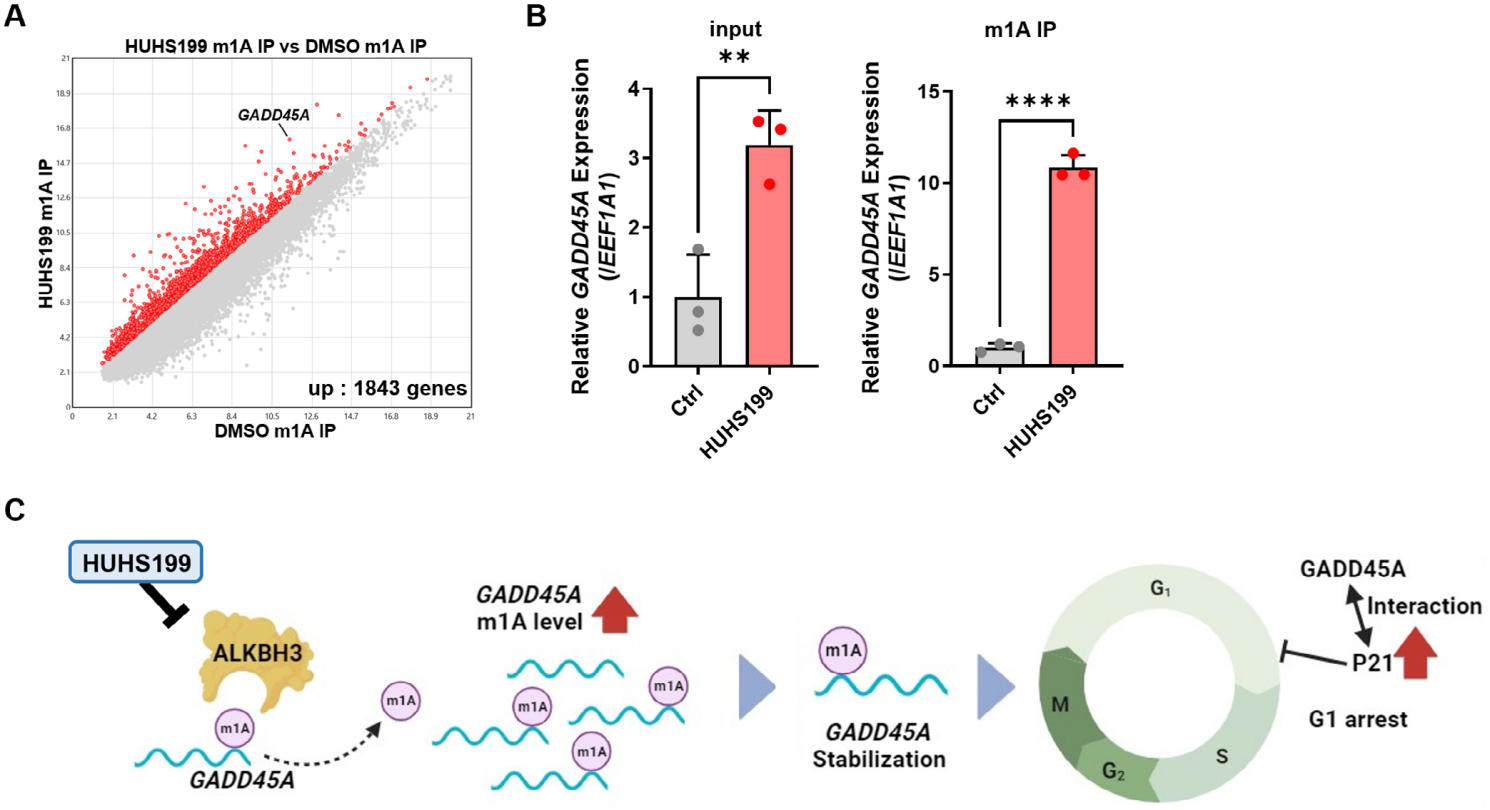
HUHS199 increases m1A levels in *GADD45A* mRNA. (A) RNA immunoprecipitation–microarray analysis using anti‐m1A antibody identified m1A‐containing genes whose expression increased in U251MG cells upon HUHS199 addition. (B) qPCR analysis of *GADD45A* mRNA expression in U251MG cells treated with HUHS199 (10 μM). Values are shown as mean ± S.D. (*n* = 3); paired *t*‐test: **p < 0.01, ****p < 0.0001. (C) Proposed mechanism by which HUHS199 induces proliferation suppression in GBM cells.

To identify m1A‐containing genes targeted by ALKBH3, HUHS199 was used as a tool compound. Immunoprecipitation using an anti‐m1A antibody followed by RNA microarray analysis identified 1843 m1A‐containing genes with increased m1A levels upon HUHS199 treatment compared with the DMSO control (Figure 5A). Among these, growth arrest and GADD45A were identified as the m1A‐containing genes that exhibited a particularly significant increase in expression. GADD45A is involved in DNA damage response and cell cycle regulation; it is known to suppress cell proliferation via cell cycle arrest (Wang et al. 1999). qPCR analysis of *GADD45A* expression in HUHS199‐treated U251MG cells revealed a significant increase in expression in the input and m1A immunoprecipitated samples (Figure 5B). Furthermore, *GADD45A* mRNA expression increased upon HUHS199 treatment of LN229 cells (Figure S6A). Interestingly, in LN229 cells, HUHS199 addition was confirmed to prolong the half‐life of *GADD45A* mRNA (Figure S6B). This finding suggests that the HUHS199‐mediated inhibition of m1A demethylation of *GADD45A* mRNA enhances RNA stabilization. A schematic representation of the mechanism of action of HUHS199 in GBM cells is shown in Figure 5C. HUHS199 inhibited the RNA demethylase activity of ALKBH3, thereby increasing the m1A levels of *GADD45A*, one of its substrates. This increase enhances *GADD45A* stability and, through interaction with p21, induces G1 phase cell cycle arrest in GBM cells.

## 4 Discussion

In this study, we demonstrate for the first time that the DNA/RNA demethylase ALKBH3 is a potential therapeutic target for GBM, the most malignant and prognostically poor primary brain tumor. Based on these findings, we developed HUHS199, a highly potent ALKBH3 inhibitor, and showed that it suppressed GBM cell proliferation by inducing G1 phase cell cycle arrest. Furthermore, m1A on *GADD45A* was identified as a candidate substrate of the ALKBH3 demethylase.

More than 170 types of chemical modifications, including methylation, hydroxymethylation, and acetylation, have been identified in cellular RNA (Boccaletto et al. 2022). Among these, methylated modifications such as m6A (He et al. 2019; Deng et al. 2022), m1A (Li et al. 2022), and m3C (Mao et al. 2021) are the most extensively studied. These methylated RNA have distinct biological functions. For example, m6A, the most abundant methylated modification in eukaryotic mRNA, is involved in precursor mRNA processing and promotes mRNA transport (Camper et al. 1984; Finkel and Groner 1983). The role of m6A in tumors, particularly in GBM, has been extensively studied, with many reports linking it to tumor stemness, cell proliferation, and treatment resistance to temozolomide. FTO and ALKBH5, which function as m6A demethylases on RNA, have been recognized as oncogenic factors (Zhang et al. 2017). Cui et al. demonstrated that a specific inhibitor of FTO reduced the tumorigenic potential of GBM stem cells (Cui et al. 2017), whereas Su et al. confirmed that reduced FTO activity inhibited GBM cell proliferation and survival by regulating Myc expression (Su et al. 2018). ALKBH5 is overexpressed in GBM stem cells and is associated with poor prognosis in patients with GBM (Kowalski‐Chauvel et al. 2020). In contrast, m1A is known to regulate diverse RNA metabolic processes, including RNA structure, stability, and translation efficiency (Zhao et al. 2017). Although m1A dysregulation has been implicated in tumor progression and malignancy (Jin et al. 2022; Shi et al. 2020), its molecular significance in GBM remains poorly understood. In this study, we confirm that the m1A demethylase ALKBH3 is highly expressed in tumor regions compared with that in non‐tumor regions in GBM postoperative specimens, whereas m1A levels are reduced. Furthermore, analysis of the TCGA database showed that high expression of ALKBH3 was associated with poorer prognosis in GBM, particularly in proneural and mesenchymal subtypes. Proneural–mesenchymal transition reflects the malignant progression and recurrence of GBM (Lai et al. 2023), suggesting that ALKBH3 contributes to the malignant transformation of GBM. Based on these findings, we proposed ALKBH3 as a molecular target in GBM and developed HUHS199 as a potent ALKBH3 enzyme inhibitor with growth‐suppressive effects on GBM cells.

ALKBH3 oxidatively demethylates m1A and m3C in RNA and ssDNA substrates (Trewick et al. 2002; Aas et al. 2003; Ougland et al. 2004). In this study, in vitro demethylase activity assays showed that HUHS199 inhibited ALKBH3‐mediated m1A demethylation to a similar extent in DNA and RNA substrates. However, immunocytochemical staining with an anti‐m1A antibody in GBM cells revealed increased m1A signals in the cytoplasm following HUHS199 treatment, which were significantly attenuated by RNase treatment. Given that ALKBH3 is primarily localized in the cytoplasm (Chen et al. 2019), these findings suggest that HUHS199 predominantly inhibits ALKBH3 activity on RNA rather than on DNA in GBM cells, thus resulting in increased m1A levels.

Recent studies have identified RNA types demethylated by ALKBH3. Chen et al. reported that ALKBH3 demethylated m1A58, m3C32, and m3C47 on tRNA (Chen et al. 2019), with m1A58 demethylation specifically inducing tRNA‐derived RNA (tDR) production. Additionally, m1A‐modified mRNAs such as the cytokine macrophage colony‐stimulating factor *CSF‐1* (Woo and Chambers 2019), the glycolytic key enzyme *aldolase A* (Deng et al. 2025) in breast cancer, and *AURORA A* (Kuang et al. 2022), a major inhibitory factor in ciliogenesis, have been identified as ALKBH3 targets in other cancer types. However, no ALKBH3‐targeted m1A‐modified mRNA has been identified in GBM. In this study, we identified m1A on *GADD45A* as a candidate substrate of the demethylase ALKBH3 using HUHS199 treatment combined with microarray analysis of immunoprecipitants using anti‐m1A antibodies. GADD45A is induced by DNA damage responses and stress signals and plays a key role in cell cycle regulation. Overexpression of GADD45 in human primary fibroblasts induces cell cycle arrest at the G2/M phase (Wang et al. 1999). In human small‐cell lung cancer cells, the induced expression of GADD45A suppressed cell proliferation and increased cell accumulation in the G1 phase of the cell cycle (Zhang et al. 2001). Mechanistically, GADD45A induces G2/M cell cycle arrest by binding to CDC2–cyclin B1, leading to the dissociation of the CDC2–cyclin B1 complex and inhibition of CDC2 kinase activity (Vairapandi et al. 2002). However, the mechanism underlying GADD45A‐induced G1 phase arrest remains unclear. Evidence suggests that GADD45A interacts with p21 (Kearsey et al. 1995; Zhao et al. 2000) and induces G1 cell cycle arrest through regulation of CDK4/6–cyclin D activity via p21 (Abbas and Dutta 2009; Karimian et al. 2016). In this study, HUHS199 treatment induced G1 phase arrest and suppressed the proliferation of U251MG, LN229, and U87MG cells. Notably, a marked increase in p21 expression was observed at mRNA and protein levels in HUHS199‐treated GBM cells. An increase in *p21* mRNA expression was also observed in *ALKBH3*‐knockdown GBM cells; the finding that *p21* was induced by *ALKBH3* knockdown and HUHS199 treatment supported the conclusion that HUHS199 acted by targeting ALKBH3. To confirm the effect of increased GADD45A expression on GBM cells, we evaluated the proliferation of GBM cells overexpressing GADD45A; however, no significant inhibitory effect on cell proliferation was observed (data not shown). These findings indicate that GADD45A overexpression alone is not sufficient to suppress GBM cell proliferation. Therefore, we hypothesize that HUHS199 induces G1 cell cycle arrest and suppresses proliferation in GBM cells via increased expression of GADD45A and p21, although further analysis is required to confirm this mechanism.

Multiple factors, including the RNA three‐dimensional structure determined by RNA–protein and RNA–RNA interactions, are involved in the post‐transcriptional regulation of mRNA expression. Recent studies have also highlighted that chemical modifications of RNA contribute to the regulation of RNA stability and degradation (Roundtree et al. 2017). For instance, the ALKBH3‐mediated m1A demethylation of *CSF‐1* mRNA prolongs its half‐life in breast and ovarian cancer cells (Woo and Chambers 2019), whereas the loss of ALKBH3 suppresses m1A demethylation of *Aurora A* mRNA, resulting in its degradation (Kuang et al. 2022). These prior findings suggest that ALKBH3‐mediated m1A demethylation generally contributes to target mRNA stabilization. However, in this study, we found that the stability of *GADD45A* mRNA increased in LN229 cells treated with HUHS199. This finding indicates that m1A demethylation by ALKBH3 does not necessarily lead to mRNA stabilization; its effect may vary depending on the target gene, m1A‐recognized reader protein, or cell type.

In conclusion, this study demonstrates that HUHS199, a patented compound that inhibits ALKBH3 enzyme activity, suppresses the m1A demethylation of *GADD45A* mRNA and inhibits GBM cell proliferation, likely through cooperative regulation of GADD45A and p21. These findings highlight the involvement of ALKBH3 in mRNA stability and cell cycle control via post‐transcriptional modifications. The cytotoxicity of HUHS199 in the normal human cell line was approximately tenfold lower than that in GBM cells. These results indicate that superiorly selective and promising ALKBH3 inhibitors can be developed through derivative synthesis. Furthermore, in a mouse xenograft model, HUHS199 administration (15 mg/kg, tail vein injection) did not cause weight loss (data not shown), suggesting a favorable safety profile. Therefore, HUHS199 represents a promising first‐in‐class molecularly targeted therapeutic candidate against GBM that targets the inhibitory activity of ALKBH3. Further in vivo validation of its efficacy and safety is warranted to support the development of novel ALKBH3‐targeted treatment strategies for GBM.

## Author Contributions

**Manami Yamada:** conceptualization, visualization, resources, writing – original draft. **Hiroaki Hase:** supervision, writing – review and editing. **Kaori Kitae:** resources, investigation. **Yuko Ueda:** resources, investigation. **Tatsuhiko Furukawa:** resources, writing – review and editing. **Toshiya Morie:** resources, writing – review and editing. **Shuntaro Aoi:** investigation. **Ryosuke Hanaya:** resources, writing – review and editing. **Nayuta Higa:** resources, writing – review and editing. **Kazutake Tsujikawa:** writing – review and editing, project administration, funding acquisition, conceptualization. **Honoka Kitamura:** resources, investigation.

## Funding

This work was supported by MEXT (24K02179), BINDS (JP26ama121054) and JST SPRING (JPMJSP2138).

## Conflicts of Interest

The authors declare no conflicts of interest.

## Supporting information


**Figure S1:** Overall survival of patients with GBM classified by transcriptionally defined subtypes (Classical, Mesenchymal, and Proneural).

**Data S1:** Supporting Information.

**Figure S2:** (A) *ALKBH3* mRNA levels were analyzed by qPCR in control siRNA‐transfected LN229 cells (siControl) or ALKBH3‐knockdown LN229 cells (siALKBH3). Values are shown as mean ± S.D. (*n* = 3); one‐way ANOVA post hoc Bonferroni's test: ***p < 0.001. (B) Western blot analysis of whole cell lysates prepared from control LN229 cells (siControl) or ALKBH3‐knockdown LN229 cells (siALKBH3) using anti‐ALKBH3 and anti‐EEF1A1 antibodies. Representative images from three independent experiments are shown. (C) Evaluation of proliferative potential of ALKBH3‐knockdown LN229 cells by real‐time cell analysis system. The data shows representative cell proliferation curves from duplicate experiments. (D) qRT‐PCR analysis of *p21* mRNA levels in ALKBH3‐knockdown LN229 cells. Values are shown as mean ± S.D. (*n* = 3); one‐way ANOVA post hoc Bonferroni's test: **p < 0.01.

**Figure S3:** (A) Compound synthesis design strategy for the novel ALKBH3 enzyme activity inhibitor HUHS199. (B) Synthesis pathway of HUHS199. (C) Comparison of the inhibitory effects of HUHS199 and HUHS015 on ALKBH3 demethylase activity using the m1A‐containing DNA oligonucleotide as a substrate.

**Figure S4:** (A) Inhibitory effect of HUHS199 on proliferation of LN229 and U87MG cells. HUHS199 and TMZ were added to the GBM cell lines and cultured for 2 days. Values are shown as mean ± S.D. (*n* = 3); one‐way ANOVA post hoc Bonferroni's test: **p < 0.01, ***p < 0.001, ****p < 0.0001, TMZ: Temozolomide. (B) The effect of HUHS199 on the proliferation of PSVK1 cells. Values are shown as mean ± S.D. (*n* = 3); one‐way ANOVA post hoc Bonferroni's test: *p < 0.05, ****p < 0.001, (C) Cell cycle analysis of LN229 and U87MG cells upon HUHS199 addition by flow cytometry. Values are shown as mean ± S.D. (*n* = 3); one‐way ANOVA post hoc Bonferroni's test: *p < 0.05, **p < 0.01, ***p < 0.001. (D) qPCR analysis of *p21* mRNA expression in LN229 cells treated with HUHS199. Values are shown as mean ± S.D. (*n* = 3); one‐way ANOVA post hoc Bonferroni's test: **p < 0.01. (E) Western blot analysis of whole cell lysates prepared from LN229 cells treated with HUHS199 using anti‐p21 and anti‐EEF1A1 antibodies. (E) Original uncropped Western blot scans.

**Figure S5:** (A) Evaluation of m1A levels in LN229 cells upon HUHS199 addition by flow cytometry. LN229 cells treated with control or HUHS199 were fixed and stained with anti‐m1A antibody. The average fluorescence intensity measured within the cells by flow cytometry was expressed as mean fluorescence intensity (MFI). Values are shown as mean ± S.D. (*n* = 3); one‐way ANOVA post hoc Bonferroni's test: *p < 0.05. (B) Evaluation of intracellular m1A levels in LN229 cells upon HUHS199 (33 μM) addition by immunocytochemistry. Each profile (red: m1A, blue: DAPI) shows the fluorescence intensity distribution along the yellow line indicated in the image. Scale bar (white): 20 μm. Representative images from three independent experiments are shown.

**Figure S6:** (A) qPCR analysis of *GADD45A* mRNA expression in LN229 cells upon HUHS199 (10 μM) addition. Values are shown as mean ± S.D. (*n* = 3); unpaired *t*‐test: **p < 0.01. (B) Analysis of *GADD45*A mRNA half‐life in LN229 cells upon HUHS199 (10 μM) addition. Actinomycin D was added at a concentration of 1 μg/mL. Data are presented as mean ± S.D. from three representative experiments.

## Data Availability

The data that support the findings of this study are available from the corresponding author upon reasonable request and with a data‐sharing agreement.
