# Supplementary material for: An ALKBH3 Inhibitor HUHS199 Suppresses Proliferation of Glioblastoma Cells by Targeting 1‐Methyladenosine on GADD45A mRNA
Source: Chem Biol Drug Des. 2026 Sep 1;108(3):e70385. doi: 10.1111/cbdd.70385 (PMC13534923; doi:10.1111/cbdd.70385)
Supplement: Supplementary file 1 [file CBDD-108-e70385-s006.docx]

**Supplementary Methods**

**Synthesis of N-cyclohexyl-2-(3-hydroxy-4,5,6,7-tetrahydro-2H-indazol-2-yl)-1H-benzo[d]imidazole-5-sulfonamide (HUHS199)**

To a solution of 2-chloro-N-cyclohexyl-1H-benzo[d]imidazole-5-sulfonamide (compound 1, supplemental figure 1B) (Lin et al., 2016) (8.1 g, 35.0 mmol) in DMF (81 mL), triethylamine (7.1 g, 70.0 mmol) and cyclohexylamine (6.9 g, 70.0 mmol) were added under ice cooling. After stirring for 16 h at room temperature, the reaction mixture was added to 1 N HCl (160 mL) on ice, and the precipitate was collected by filtration. The precipitate was washed with water and dried in vacuo to obtain N-cyclohexyl-2-oxo-2,3-dihydro-1H-benzo[d]imidazole-5-sulfonamide (2) (6.9 g, 67%). 1 H-NMR (400 MHz, DMSO-d6) δ 0.85-1.15 (5 H, m), 1.34-1.43 (1 H, m), 1.44-1.60 (4 H, m), 2.75-2.90 (1 H, m), 7.04 (1 H, d, J = 8.4 Hz), 7.32 (1 H, d, J = 1.4 Hz), 7.42 (1 H, dd, J = 8.4 and 1.4 Hz), 7.44 (1 H, d, J = 7.6 Hz), 10.96 (1 H, s), 11.04 (1 H, s).

Compound 2 (2.0 g, 6.8 mmol), phosphoryl chloride (25 mL) was stirred at 120℃ for 4 h. After cooling to ambient temperature, 1 N NaOH solution was added to the mixture to pH8. The aqueous phase was extracted with ethyl acetate, and the organic phases were washed with water and brine, dried over anhydrous MgSO4, filtered, and concentrated under reduced pressure. The crude product was purified by silica gel column chromatography (n-hexane/ethyl acetate = 1 / 2) to afford 2-chloro-N-cyclohexyl-1H-benzo[d]imidazole-5-sulfonamide (compound 3) (626 mg, 30%) as a pale-yellow solid.

LC-MS RT: 2.42 min 314 ［M+1］

Compound 3 (430 mg, 1.37 mmol) and hydrazine monohydrate (1.37 g, 27.4 mmol) were stirred at 120℃ for 12 h. After cooling to the ambient temperature, water was added to the reaction mixture. The precipitate was collected via filtration. The precipitate was washed with water and dried in vacuo to afford N-cyclohexyl-2-hydrazineyl-1H-benzo[d]imidazole-5-sulfonamide (compound 4) (340 mg, 80%) as a white solid. LC-MS RT: 1.12 min 310［M+1］

Compound 4 (340 mg, 1.1 mmol) and ethyl 2 -oxocyclohexanecarboxylate (187 mg, 1.1 mmol) were stirred in acetic acid (2 mL) for 16 h at room temperature. The reaction mixture was poured into water and stirred at the ambient temperature. The precipitate was collected via filtration and washed with water. The precipitate was recrystallized from a mixture of ethanol/water (1/1) and dried in vacuo to afford N-cyclohexyl-2-(3-hydroxy-4,5,6,7-tetrahydro-2H-indazol-2-yl)-1H-benzo[d]imidazole-5-sulfonamide (HUHS199, N-cyclohexyl-2-(3-hydroxy-4,5,6,7-tetrahydro-2H-indazol-2-yl)-1H-benzo[d]imidazole-5-sulfonamide （HUHS199, supplemental figure 1A) (220 mg, 48%). 1 H-NMR (400 MHz, DMSO-d6) δ 0.92-1.18 (5 H, m), 1.35-1.45 (1 H, m), 1.46-1.60 (4 H, m), 1.63-1.78 (4 H, m), 2.22 (2 H, t, J = 6.0 Hz), 2.51 (2 H, t, J = 6.0 Hz), 2.90 (br. s), 7.51 (1 H, d, J = 6.8 Hz), 7.61 (1 H, dd, J = 8.4 and 1.6 Hz), 7.65 (1 H, d, J = 8.4 Hz), 7.96 (1 H, br. s).

LC-MS RT:　2.28 min　416［M+1］Melting point: 175–177 ℃.

**Chemistry**

All the reactions were performed in dry solvents under an argon atmosphere. Unless otherwise noted, all the substrates and solvents were purchased from commercial sources and used without further purification. Flash column chromatography was performed with 40–50 µm Silica Gel 60N (Kanto Chemical Co., Inc., Tokyo, Japan). Melting points were measured on YanacoMP-S3.1H NMR spectra were recorded on a JEOL ECA 500 spectrometer (1H: 500 MHz) at room temperature in DMSO-d6 as a solvent with chemical shifts reported in δ (ppm) relative to the residual solvent signal (DMSO-d6: δ = 2.50 ppm), and coupling constants (J) are in hertz (Hz). Multiplicities were reported using the following abbreviations:　s = singlet, d = doublet, m = multiplet, dd = doublet of doublets, and br=broad. LC-MS analyses were performed using a Waters ACQUITY UPLC H-Class/QDa system. The mobile phases were [A] 0.1% formic acid-containing aqueous solution and [B] acetonitrile. The column was a 1.7 µm, 2.1 × 50 mm ACQUITY UPLC BEH C18 column. Samples were run on a 3 min gradient from 10 to 95% organic at 0.5 mL/min. PDA was used to analyze the wavelength range 190 of 400 nm.

**Supplementary Figures**

**Fig. S1**

Overall survival of patients with GBM classified by transcriptionally defined subtypes (Classical, Mesenchymal, and Proneural).

**Fig. S2**

(A) *ALKBH3* mRNA levels were analyzed by qPCR in control siRNA-transfected LN229 cells (siControl) or ALKBH3-knockdown LN229 cells (siALKBH3). Values are shown as mean ± S.D. (n=3); one-way ANOVA post hoc Bonferroni's test: ***p<0.001. (B) Western blot analysis of whole cell lysates prepared from control LN229 cells (siControl) or ALKBH3-knockdown LN229 cells (siALKBH3) using anti-ALKBH3 and anti-EEF1A1 antibodies. Representative images from three independent experiments are shown. (C) Evaluation of proliferative potential of ALKBH3-knockdown LN229 cells by real-time cell analysis system. The data shows representative cell proliferation curves from duplicate experiments. (D) qRT-PCR analysis of *p21* mRNA levels in ALKBH3-knockdown LN229 cells. Values are shown as mean ± S.D. (n=3); one-way ANOVA post hoc Bonferroni’s test: **p<0.01.

**Fig. S3**

(A) Compound synthesis design strategy for the novel ALKBH3 enzyme activity inhibitor HUHS199. (B) Synthesis pathway of HUHS199. (C) Comparison of the inhibitory effects of HUHS199 and HUHS015 on ALKBH3 demethylase activity using the m1A-containing DNA oligonucleotide as a substrate.

**Fig. S4**

(A) Inhibitory effect of HUHS199 on proliferation of LN229 and U87MG cells. HUHS199 and TMZ were added to the GBM cell lines and cultured for 2 days. Values are shown as mean ± S.D. (n=3); one-way ANOVA post hoc Bonferroni’s test: **p<0.01, ***p<0.001, ****p<0.0001, TMZ: Temozolomide. (B) The effect of HUHS199 on the proliferation of PSVK1 cells. Values are shown as mean ± S.D. (n=3); one-way ANOVA post hoc Bonferroni’s test: *p<0.05, ****p<0.001, (C) Cell cycle analysis of LN229 and U87MG cells upon HUHS199 addition by flow cytometry. Values are shown as mean ± S.D. (n=3); one-way ANOVA post hoc Bonferroni’s test: *p<0.05, **p<0.01, ***p<0.001. (D) qPCR analysis of *p21* mRNA expression in LN229 cells treated with HUHS199. Values are shown as mean ± S.D. (n=3); one-way ANOVA post hoc Bonferroni’s test: **p<0.01. (E) Western blot analysis of whole cell lysates prepared from LN229 cells treated with HUHS199 using anti-p21 and anti-EEF1A1 antibodies. (E) Original uncropped Western blot scans.

**Fig. S5**

(A) Evaluation of m1A levels in LN229 cells upon HUHS199 addition by flow cytometry. LN229 cells treated with control or HUHS199 were fixed and stained with anti-m1A antibody. The average fluorescence intensity measured within the cells by flow cytometry was expressed as mean fluorescence intensity (MFI). Values are shown as mean ± S.D. (n=3); one-way ANOVA post hoc Bonferroni’s test: *p<0.05. (B) Evaluation of intracellular m1A levels in LN229 cells upon HUHS199 (33 µM) addition by immunocytochemistry. Each profile (red: m1A, blue: DAPI) shows the fluorescence intensity distribution along the yellow line indicated in the image. Scale bar (white): 20 µm. Representative images from three independent experiments are shown.

**Fig. S6**

(A) qPCR analysis of *GADD45A* mRNA expression in LN229 cells upon HUHS199 (10 µM) addition. Values are shown as mean ± S.D. (n=3); unpaired t-test: **p<0.01. (B) Analysis of *GADD45*A mRNA half-life in LN229 cells upon HUHS199 (10 µM) addition. Actinomycin D was added at a concentration of 1 µg/mL. Data are presented as mean ± S.D. from three representative experiments.
